# CYP1A1 MspI polymorphism and acute myeloid leukemia risk: meta-analyses based on 5018 subjects

**DOI:** 10.1186/1756-9966-31-62

**Published:** 2012-07-30

**Authors:** Wenlei Zhuo, Liang Zhang, Yan Wang, Bo Zhu, Zhengtang Chen

**Affiliations:** 1 Institute of Cancer, Xinqiao Hospital, Third Military Medical University, Chongqing, China; 2 Department of Environmental Hygiene, College of Preventive Medicine, Third Military Medical University, Chongqing, China; 3 Institute of Respiratory Diseases,Xinqiao Hospital, Third Military Medical University, Chongqing, China

**Keywords:** CYP1A1 MspI, Acute myeloid leukemia, Malignancy, Susceptibility, Meta-analysis, Polymorphism

## Abstract

**Background:**

Evidence indicates that CYP1A1 MspI polymorphism might be a possible risk factor for several malignancies. A growing body of literature has been devoted to the association of CYP1A1 MspI polymorphism with acute myeloid leukemia (AML). However, the results remain conflicting. The aim of the present study was to derive a more precise estimation of the relationship.

**Methods:**

Meta-analyses assessing the association of CYP1A1 MspI variation with AML were conducted and subgroup analyses on ethnicity and age groups were further performed. Eligible studies were identified for the period up to May 2012.

**Results:**

A total of ten case–control studies including 1330 cases and 3688 controls were selected for analysis. The overall data failed to indicate a significant association of CYP1A1 MspI polymorphism with AML risk (C vs T: OR = 1.13; 95%CI = 0.87-1.48; CC vs TT: OR = 1.72; 95%CI = 0.99-3.01; CC + TC vs TT: OR = 1.16; 95%CI = 0.86-1.55). In subgroup analysis stratified by ethnicity, significant AML risk was shown among Asians (CC + TC vs TT: OR = 1.33; 95%CI = 1.09-1.62) but not Caucasians or mixed races. In subgroup analysis regarding age groups, no associations were observed in either the childhood AML or the adult AML subgroups.

**Conclusion:**

The results of the present study suggested that CYP1A1 MspI polymorphism might be a risk factor for AML among Asians. Further investigations are needed to confirm the conclusions.

## Introduction

Acute myeloid leukemia (AML), also known as acute nonlymphocytic leukemia (ANLL), is the most common acute leukemia mostly affecting adults, characterized by the rapid growth of abnormal white blood cells in the bone marrow and impaired production of normal blood cells. The mechanisms for AML genesis are still rarely understood. Evidence suggests that radiation, smoking, obesity and exposure to chemical carcinogens are considered as its possible risk factors [1]. Nevertheless, AML only develops in a small proportion of people exposed to these environmental and lifestyle risk factors, indicating that the host genetic background might play a critical role in its genesis.

Several genetic polymorphisms have been determined as possible risk factors for leukemia by meta-analyses. Variations of GSTM1, GSTT1, MTHFR C677T and XRCC1 Arg399Gln have been indicated to raise leukemia susceptibility [2-4]. Nevertheless, polymorphic MTR A2756G has been shown to decrease acute leukemia risk [5]. Therefore, different genetic polymorphisms might exert different effects on leukemia risk. Nevertheless, only a few gene polymorphisms associated with leukemia susceptibility have been identified to date.

Recent evidence indicates that carcinogen-metabolizing genes might play critical roles in determining individual susceptibility to cancers [6]. Susceptibility to cancer is determined by the activation of enzymes involved in carcinogen activation or deactivation. Polymorphisms in these genes encoding the enzymes, possibly by altering their functions, might increase or decrease carcinogen activation/detoxification and modulate DNA repair process. Cytochrome P450 (CYP) enzymes catalyze Phase I metabolism reaction. Cytochrome P450 1A1 (CYP1A1) is a member of the CYP family that participates in the metabolism of xenobiotics and endogenous compounds, particularly polycyclic aromatic hydrocarbons (PAHs) such as benzo[a]pyrene in smoke [7]. A commonly studied single nucleotide polymorphism (SNP) in the CYP1A1 gene has been indicated to associate with cancer susceptibility. The SNP locates at nucleotide 3801 in the 3’ non-coding region containing a single T to C base substitution that results in a polymorphic restriction site for the MspI enzyme (MspI or CYP1A1*2A polymorphism, rs4646903). The MspI restriction site polymorphism results in three genotypes: a predominant homozygous m1 allele without the MspI site (type A, TT), the heterozygote (type B, TC) and a homozygous rare m2 allele with the MspI site (type C, CC) [8].

Published studies devoted to the relationship between CYP1A1 MspI polymorphism and AML risk have generated controversial results. The issue of whether CYP1A1 MspI polymorphism is a risk factor for AML remains uncertain. Therefore, in this study, we aimed to perform a quantitative meta-analysis that increased statistical power to generate more confidential results.

## Materials and methods

### Literature search strategy

We carried out a search in the Medline, EMBASE, OVID, Sciencedirect, and Chinese National Knowledge Infrastructure (CNKI) without a language limitation, covering all publications published up to May 2012, with a combination of the following keywords: *Cytochrome P450 1A1, CYP1A1, T3801C, MspI, acute myeloid leukemia, acute nonlymphocytic leukemia, hematology,malignancy, neoplasm, cancer, variation* and *polymorphism*. All searched studies were retrieved and the bibliographies were checked for other relevant publications. Review articles and bibliographies of other relevant studies identified were hand searched to find additional eligible studies.

### Inclusion and exclusion criteria

The following criteria were used for the literature selection: first, studies should concern the association of CYP1A1 MspI polymorphism with AML risk; second, studies must be observational studies (Case—control or cohort); third, papers must offer the size of the sample, odds ratios (ORs) and their 95% confidence intervals (CIs), the genetic distribution or the information that can help infer the results. Accordingly, the following criteria for exclusion were also utilized: first, the design and the definition of the experiments were obviously different from those of the selected articles; second, the source of cases and controls and other essential information were not offered; third, reviews and duplicated publications. After deliberate searching, we reviewed all papers in accordance with the criteria defined above for further analysis.

### Data extraction

Data were carefully extracted from all eligible publications independently by two of the authors according to the inclusion criteria mentioned above. For conflicting evaluations, an agreement was reached following a discussion. If a consensus could not be reached, another author was consulted to resolve the dispute and then a final decision was made by the majority of the votes. The extracted information was entered into a database.

### Statistical analysis

The odds ratio (OR) of CYP1A1 MspI polymorphisms and AML risk was estimated for each study. The pooled ORs were performed for an allelic contrast (C allele versus T allele), a homozygote comparison (CC versus TT) and a dominant model (CC + TC versus TT). For detection of any possible sample size biases, the OR and its 95% confidence interval (CI) to each study was plotted against the number of participants respectively. A Chi-square based Q statistic test was performed to assess heterogeneity. If the result of the Q-test was *P* >0.1, ORs were pooled according to the fixed-effect model (Mantel-Haenszel); otherwise, the random-effect model (DerSimonian and laird) was used. The significance of the pooled ORs was determined by Z-test. The Hardy-Weinberg equilibrium (HWE) was assessed by Fisher’s exact test. Publication bias was assessed by visual inspection of funnel plots [9], in which the standard error of log (OR) of each study was plotted against its log (OR). An asymmetric plot indicates a possible publication bias. The symmetry of the funnel plot was further evaluated by Egger’s linear regression test [10]. Statistical analysis was undertaken using the program STATA 11.0 software (Stata Corporation, Texas).

## Results

### Study characteristics

**Figure 1:**
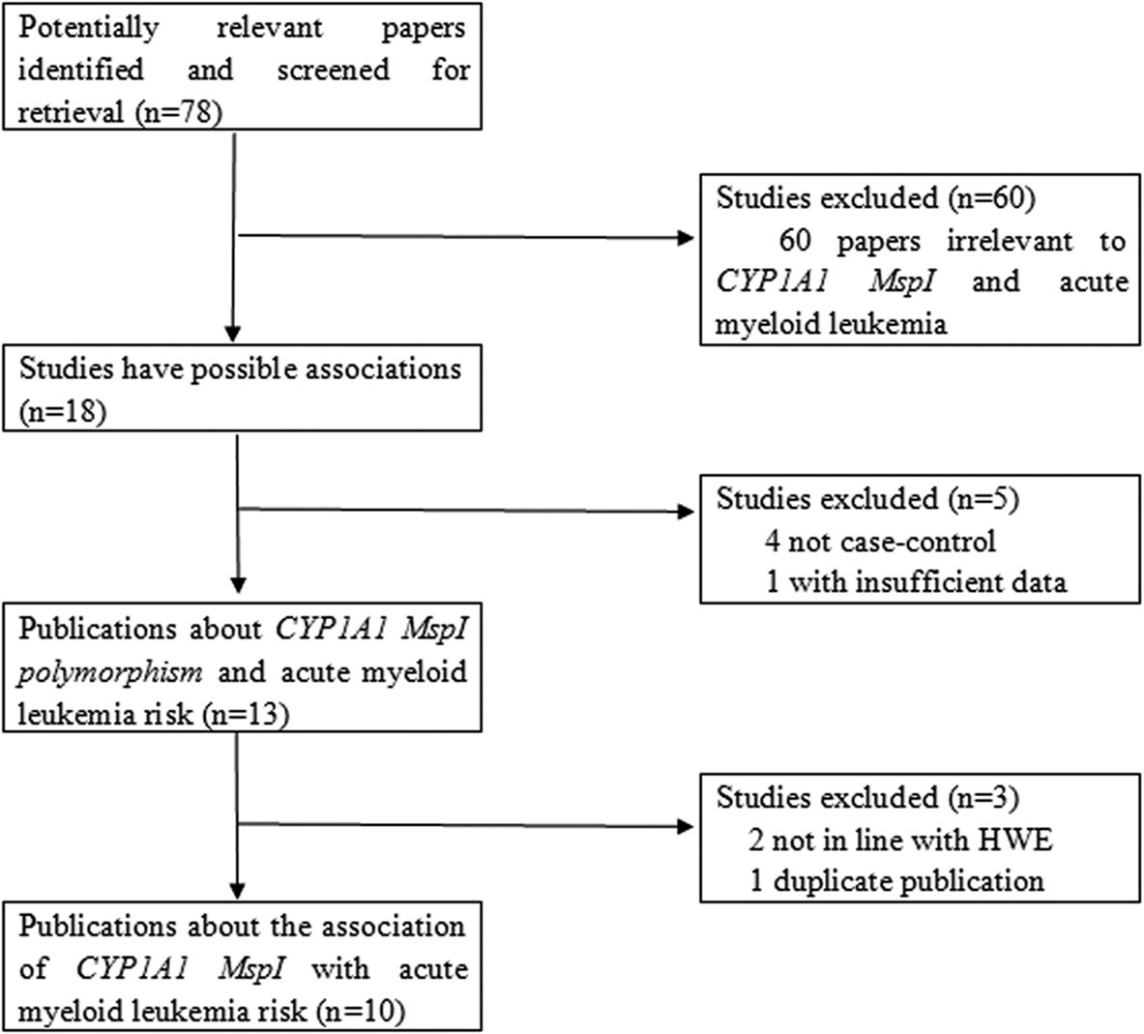
The flow diagram of included/excluded studies.

Relevant publications were retrieved and screened originally. A total of seventy-eight publications were identified, of which sixty irrelevant papers were excluded. As shown in Figure1, eighteen publications were preliminary eligible, of which four publications not being case–control studies [11-14] and one article not presenting sufficient information [15] were discarded. Next, two studies [16,17] whose genetic distributions of the control groups exhibited evident deviation from HWE were excluded. Then, one duplicate publication [18] which concerned the same research with one of the included studies [19] was further excluded. Lastly, ten case–control studies were selected for data extraction [19-28].

Of the selected publications, one was written in Chinese [24] while the remaining nine were in English. The relevant information was listed in Table1. According to this table, the first author and the number and characteristics of cases and controls for each study as well as other necessary information are presented. 

**Table 1:** Characteristics of studies included in the meta-analysis

**First Author**	**Publication Year**	**Number of Leukemia Cases (male/female)**	**Number of Controls (male/female)**	**Number of AML cases**	**Type of controls**	**Median (or mean) age, (range) year (Cases/Controls)**	**Racial decent**	**Country**
Balta	2003	33 (19/14)	185 (120/65)	33 AML	Healthy controls (PB)	8.7(1–17)/7.4(0.58-17)	Mixed	Turkey
D'Alo	2004	193 (107/86)	273(147/126)	193 AML	Healthy controls (PB)	62(19–87)/60(19–90)	Caucasian	Italy
Clavel	2005	219 (129/90)	105 (57/48)	28 AML	Non-cancer controls (age,- gender-, hospital-, ethnicity-matched; HB)	NA(0–15)/NA(0–15)	Mixed	France
Aydin-Sayitoglu	2006	249 (143/106)	140 (73/67)	50 adult AML; 44 pediatric AML	Healthy controls (PB)	Adult:33(19–75); pediatric: 7.8(2–18)/28.7(16–59)	Caucasian	Turkey
Bolufer	2007	443 (223/190)	454 (223/231)	302 AML	Healthy controls (PB)	39.48(0.8-84)/38.38(1–85)	Caucasian	Spain
Jiang	2008	98 (NA)	120 (NA)	98 AML	Healthy controls (age-, sex-matched; PB)	NA(16–68)/NA(16–68)	Asian	China
Majumdar	2008	110 (70/40)	126 (54/72)	110 AML	Healthy controls (PB)	35(4–81)/30(8–73)	Asian	India
Yamaguti	2009	133 (70/63)	133 (70/63)	133 AML	Healthy controls (PB)	47(11–89)/53(25–60)	Mixed	Brazil
Bonaventure	2012	493 (266/227)	549 (292/257)	51 AML	Healthy controls (age-, gender-matched; PB)	NA(0–15)/NA(0–15)	Caucasian	France
Kim	2012	415 (223/192)	1700 (821/879)	415 AML	Healthy controls (PB)	50.5(15–86)/52.2(20–74)	Asian	Korea

NA: not available; AML, acute myeloid leukemia; PB: population-based; HB: hospital-based.

There were four groups of Caucasians [19,21,25,26], three of Asians [20,23,24] and three of mixed races [22,27,28] in this meta-analysis. As for age groups, there were seven groups of adult AML [19,20,22-26] and four groups of childhood AML [21,25,27,28] in this study. Noticeably, the study conducted by Aydin-Sayitoglu et al… [25] involved two subgroups regarding adult AML and childhood AML, respectively.

The distributions of CYP1A1 MspI genotype as well as the genotyping methods of the included studies are presented in Table2. The genetic distributions of the control groups in all included studies were consistent with HWE.

**Table 2:** Distribution of CYP1A1 MspI genotypes among acute myeloid leukemia cases and controls included in the meta-analysis

**First Author**	**Year**	**Genotyping method**	**Cases**			**Controls**			**HWE (control)**	
			**CC**	**TC**	**TT**	**CC**	**TC**	**TT**	**Chi-squre**	**P**
Balta	2003	PCR-RFLP	0	6	20	7	35	103	2.862	> 0.05
D'Alo	2004	PCR-RFLP	0	17	161	0	42	226	1.937	> 0.05
Clavel	2005	PCR-RFLP	0	5	22	0	24	81	1.748	> 0.05
Aydin-Sayitoglu	2006	PCR-RFLP	5	24	65	4	30	106	1.049	> 0.05
Bolufer	2007	Real-time PCR	0	31	168	2	84	317	2.062	> 0.05
Jiang	2008	PCR-RFLP	19	50	29	26	50	44	2.610	> 0.05
Majumdar	2008	PCR-RFLP	30	39	41	9	51	66	0.040	> 0.05
Yamaguti	2009	PCR-RFLP	9	59	65	6	32	95	2.199	> 0.05
Bonaventure	2012	Infinium platform	2	7	41	7	87	454	1.435	> 0.05
Kim	2012	PCR-RFLP	61	219	135	263	801	636	0.170	> 0.05

### Test of heterogeneity

As shown in Table3, we analyzed the heterogeneity for the allelic contrast (C allele versus T allele), homozygote comparison (CC versus TT) and dominant model (CC + TC versus TT), respectively. Evident heterogeneities were observed for the overall data in the three genetic comparisons (C allele versus T allele: P = 0.000 for Q-test; CC versus TT: P = 0.026 for Q-test; CC + TC versus TT: P = 0.002 for Q-test). Additionally, *I*-square value is another index for the heterogeneity test [29], with value less than 25% indicating low, 25% to 50% indicating moderate, and greater than 50% indicating high heterogeneity. The *I*-square values were 71.7%, 55.9% and 65.5 for the overall data of the allelic contrast, homozygote comparison and dominant model, respectively, indicating marked heterogeneities between the studies. Hence, the random-effect models were utilized. However, when subgroup analyses regarding ethnicity and age groups were further conducted, we found loss of heterogeneities in the subgroups regarding Caucasians and childhood AML, respectively. 

**Table 3:** Main results of the pooled data in the meta-analysis

	**No. (cases/controls)**	**C allele vs T allele**			**CC vs TT**			**(CC + TC) vs TT**		
		**OR (95%CI)**	**P (OR)**	**P (Q-test)**	**OR (95%CI)**	**P (OR)**	**P (Q-test)**	**OR (95%CI)**	**P (OR)**	**P (Q-test)**
Total	1330/3688	1.13 (0.87-.1.48)	0.349	0.000	1.72 (0.99-3.01)	0.055	0.026	1.16 (0.86-1.55)	0.326	0.002
Ethnicity										
Caucasian	521/1359	0.90 (0.59-.1.37)	0.629	0.062	2.02 (0.76-5.36)	0.160	0.462	0.85 (0.57-1.26)	0.415	0.127
Asian	623/1946	1.35 (0.90-2.02)	0.150	0.004	1.77 (0.72-4.35)	0.214	0.002	*1.33 (1.09-1.62)*	*0.004*	0.382
Mixed	186/383	1.11 (0.48-2.55)	0.807	0.029	1.40 (0.28-6.90)	0.681	0.227	1.24 (0.48-3.22)	0.654	0.021
Age groups										
Adult AML	1183/2890	1.21 (0.88-1.66)	0.244	0.000	1.76 (0.94-3.30)	0.078	0.015	1.26 (0.88-1.81)	0.213	0.000
Childhood AML	147/938	1.02 (0.69-1.49)	0.938	0.620	1.78 (0.60-5.32)	0.299	0.376	0.97 (0.63-1.49)	0.877	0.856

AML, acute myeloid leukemia.

### Meta-analysis results

**Figure 2:**
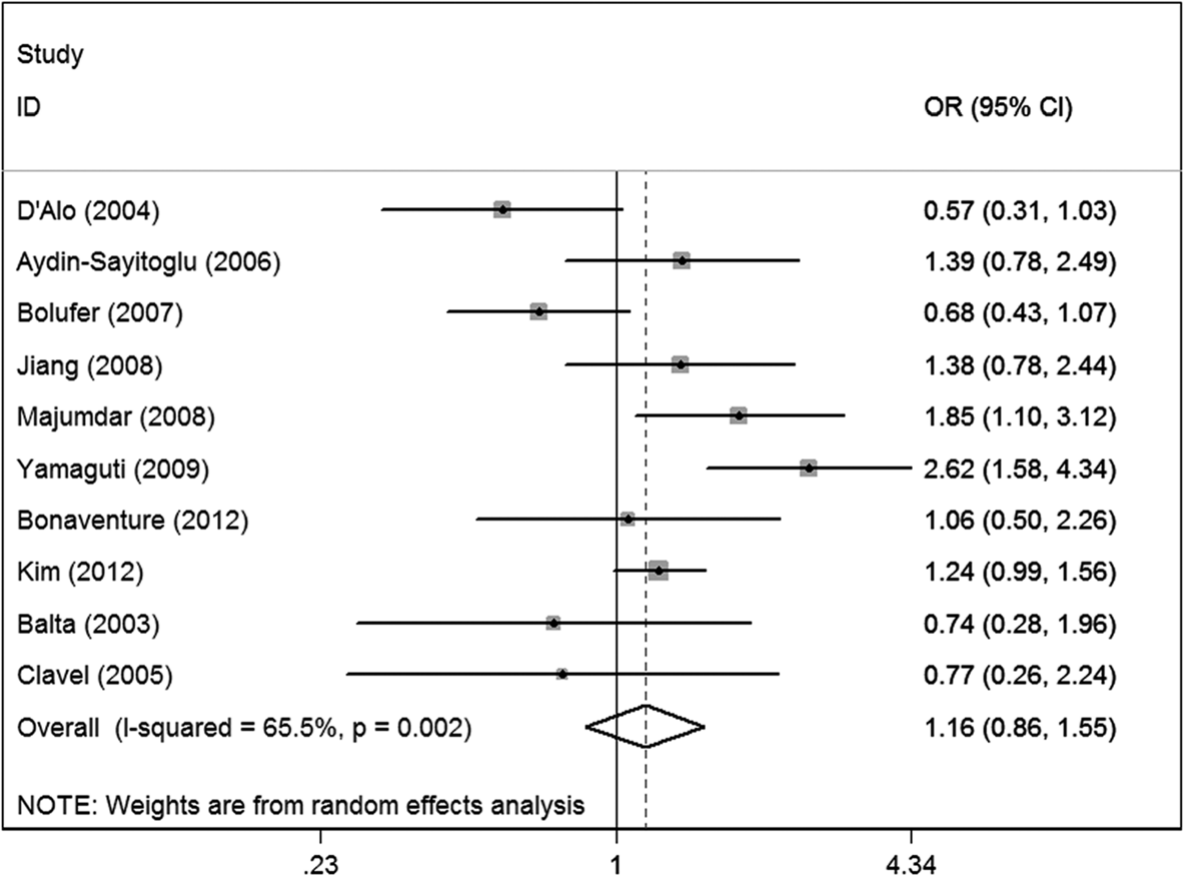
Meta-analysis for the association of acute myeloid leukemia risk with CYP1A1 MspI polymorphism for the overall data (CC + TC versus TT).

The main results of the meta-analysis were listed in Table3. For the overall data containing 1330 cases and 3688 controls, the pooled ORs for the allelic contrast, homozygote comparison and dominant model were 1.13 (95%CI = 0.87-1.48), 1.72 (95%CI = 0.99-3.01) and 1.16 (95%CI = 0.86-1.55), respectively, indicating that CYP1A1 MspI polymorphism might not have a correlation with AML risk (Figure2).

**Figure 3:**
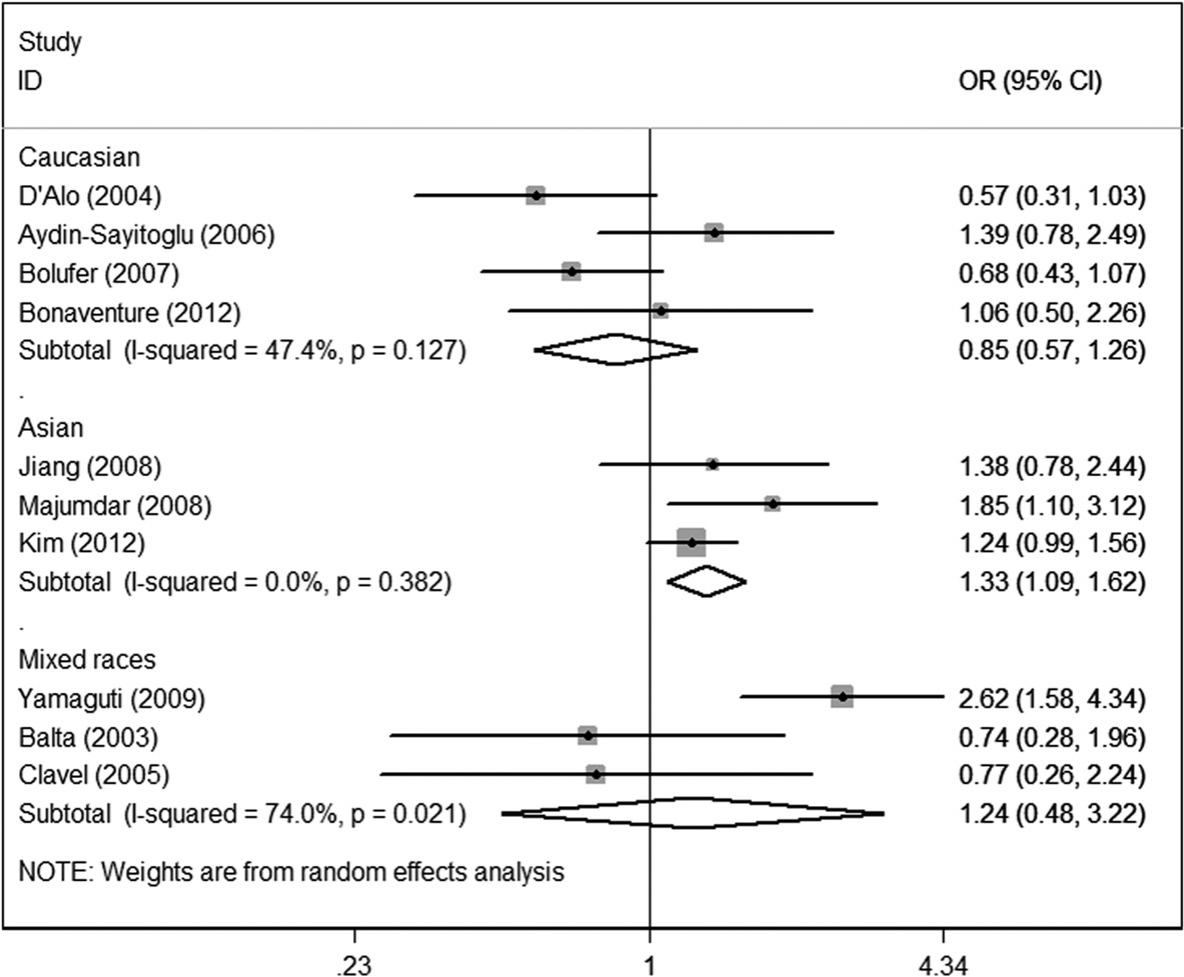
Meta-analysis for the association of acute myeloid leukemia risk with CYP1A1 MspI polymorphism (CC + TC versus TT; stratified by ethnicity).

However, in subgroup analysis according to ethnicity, increased risk was shown among Asians (OR = 1.33; 95%CI = 1.09-1.62; P = 0.382 for heterogeneity) under the dominant model, but not the allele contrast or homozygote comparison models. No increased risk could be observed among Caucasians or mixed races under the three genetic models. The data indicated that Asians who carry variant C allele might have increased AML risk relative to those who harbor wild type TT alleles. (Figure3).

**Figure 4:**
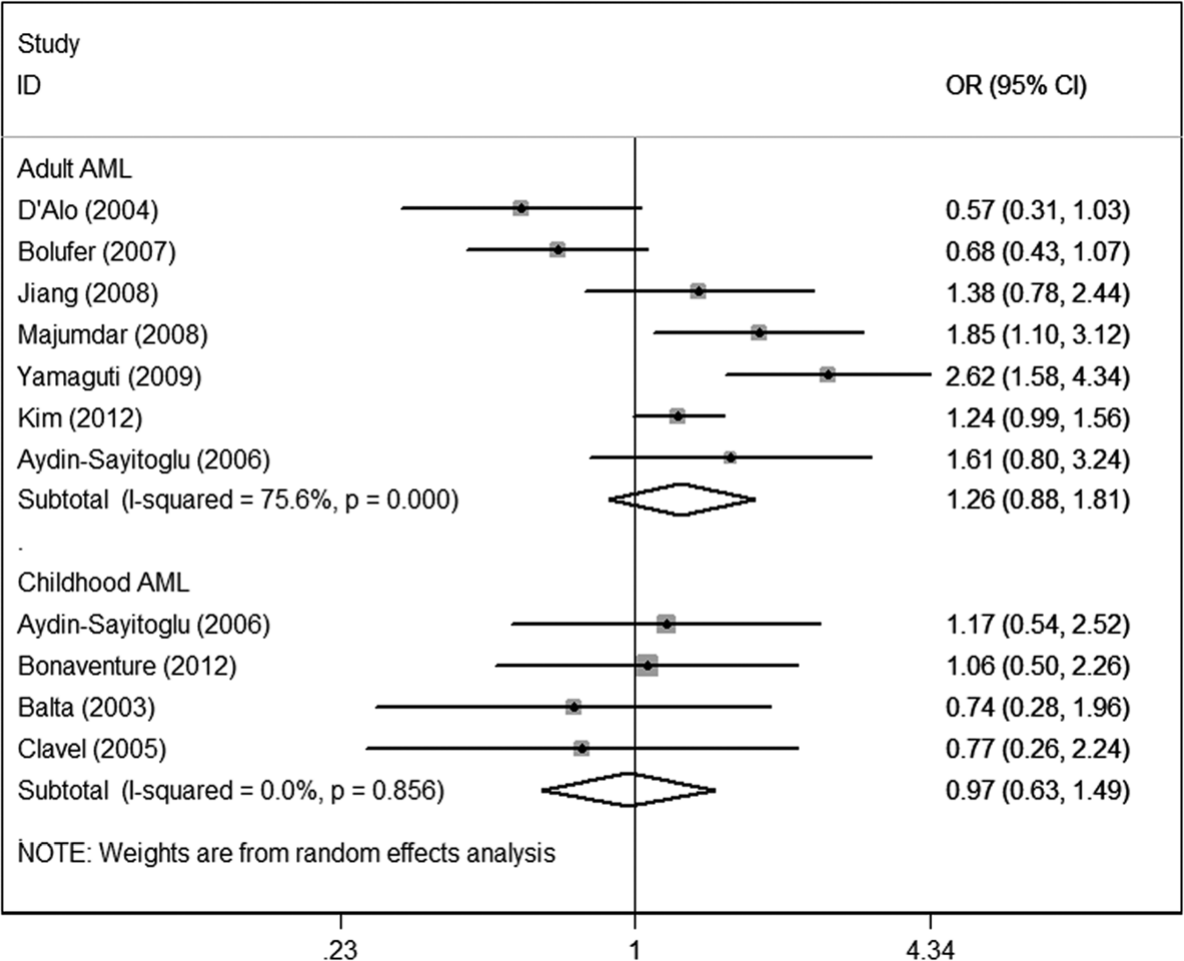
Meta-analysis for the association of acute myeloid leukemia risk with CYP1A1 MspI polymorphism stratified by age groups (CC + TC versus TT). AML, acute myeloid leukemia.

In subgroup analyses regarding age groups, no increased risk was found among either the childhood AML subgroup or the adult AML subgroup under the three genetic comparisons (Figure4).

### Sensitivity analysis

When the effect-models were changed, the significance of the overall data for the two comparisons, respectively, was not statistically altered (data not shown). Then, one-way sensitivity analysis [30] was carried out to assess the stability of the meta-analysis. The statistical significance of the results was not changed when any single study was omitted (data not shown), indicating the credibility of the results.

### Bias diagnostics

**Figure 5:**
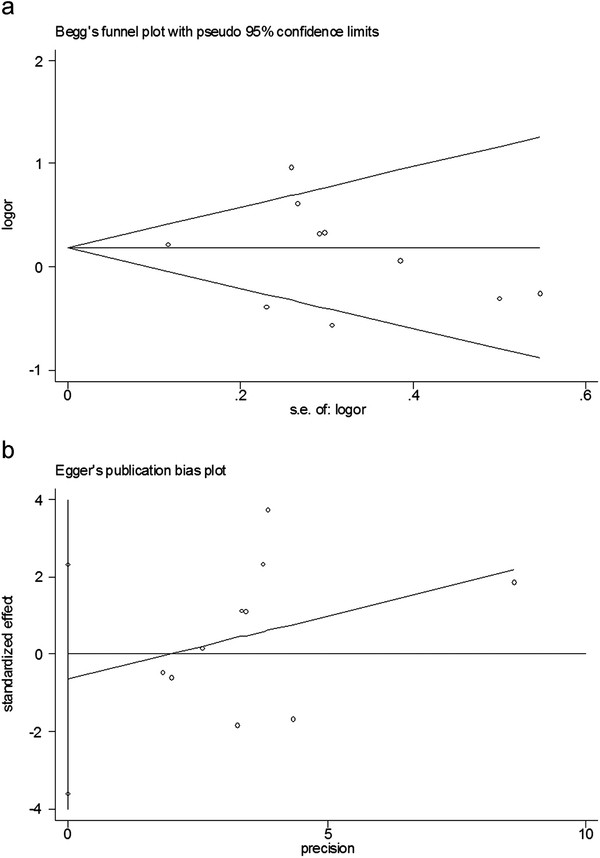
Publication bias tests for the overall data (CC + TC versus TT). (a): Funnel plot; (b) Egger’s linear regression test.

Funnel plots were created to detect possible publication bias. Then, Egger’s linear regression tests were used to assess the symmetries of the plots. The funnel plots appeared to be symmetrical for the overall data (Figure5a). Moreover, results of the Egger’s tests also indicated that the potential publication bias was not evident (Figure5b) (C allele versus T allele: t = −0.20, P > 0.05; CC versus TT: t = 0.66, P >0.05; CC + TC versus TT: t = −0.50, P >0.05).

## Discussion

For the overall data, the results showed that CYP1A1 MspI polymorphism might not have a significant correlation with AML risk. Moreover, in subgroup analyses stratified by ethnicity, the data suggested an excess AML risk among Asians but not Caucasians or mixed races.

Previously, several meta-analyses have been devoted to the association of CYP1A1 MspI polymorphism with other cancer risk. Nevertheless, the results were conflicting. CYP1A1 MspI genetic variations have been indicated to raise risk for lung cancer, cervical cancer, prostate cancer and laryngeal cancer [31-34]. However, negligible relations between polymorphic CYP1A1 MspI and gastric cancer, colorectal cancer, breast cancer and esophageal cancer risks have been found [35-38]. Therefore, polymorphism of CYP1A1 MspI might play different roles in different cancers.

As for leukemia, a recent meta-analysis by Zhang et al… [39] regarding the relations of CYP1A1 MspI polymorphism with childhood acute leukemia failed to suggest a significant association regarding childhood ANLL (AML), in line with the present study. However, in the study by Zhang et al. [39], only two studies regarding childhood AML were selected [27,28]. Another two important studies that met the inclusion criteria were ignored [21,25]. In the present meta-analysis, a total of ten studies concerning childhood AML as well as adult AML were included, which statistically increased power to assess the associations.

In subgroup analysis according to ethnicity, significant increased risk was found among Asians, but not Caucasians and mixed races. Notably, this association could be only observed in the dominant model but not the allele contrast and homozygote comparison models, indicating that Asians who bear variant C allele of CYP1A1 MspI polymorphism might have an excess AML risk compared with those who carry wild type TT alleles. Possible racial differences in presentation, treatment patterns and survival with respect to AML might exist [40]. The difference might be owing to a possible role of ethnic differences in genetic backgrounds and the environment they lived in. However, the differences might be due to chance because the limited number of included studies and small sample sizes might give rise to insufficient statistical power for detection of a minor effect. Thus, the results should be interpreted with caution because undulated risk estimation might be obtained. Further studies regarding different ethnicities with large sample sizes are needed to clarify this issue.

In the subgroup analysis stratified by age groups, no increased risk was shown among either the childhood AML or the adult AML subgroups. Evidence indicates that the etiologies of childhood AML and adult AML might be different [41]. Moreover, host genetic differences between the two age groups might exist [42]. Therefore, the possible differences regarding CYP1A1 MspI polymorphism between the two age groups should be noted in further investigations. However, the data indicated that the potential difference was not evident in the present meta-analysis.

The overall data were not stratified by source of controls because all studies concerned the population-based controls, except for one study with limited sample sizes [28]. Hospital-based controls might not be always truly representative of the general population. In addition, the population-based controls in several studies were not strictly matched to the cases. Thus, any selection bias might exist. Future studies using proper control participants with strict matching criteria and large sample sizes are important for reducing such selection bias.

In the present meta-analysis, evident between-study heterogeneities for the overall data were observed for the three comparisons, respectively, and thus, the random-effect models were utilized. In the subgroup analyses, loss of heterogeneities was also found in the subgroups regarding Caucasian and childhood AML, respectively. Though we tried to minimize the possibility of encountering heterogeneity problems by conducting a careful search of the literature and using rigorous criteria for data pooling, evident heterogeneities still existed in some of the comparisons. Therefore, heterogeneities might be multifactoral. In addition to ethnicity and age groups, other factors such as gender, source of controls, histological types and prevalence of lifestyle factors might also yield the heterogeneities.

Several limitations should be concerned in the present meta-analysis. First, the primary articles only provided data about Caucasians, Asians and mixed races. Detailed information regarding other ethnicities such as African should be concerned. Second, subgroup analyses regarding gender and other factors such as smoking, drinking and radiation exposure have not been conducted in the present study because relevant information was insufficient in the primary articles. Third, only studies written in English and Chinese were included in this meta-analysis. Any selection bias should be noted. Furthermore, although the meta-analysis in this study is suggestive, high heterogeneity and lack of significant association in any genetic model among Caucasian and Mixed subgroups or age subgroups observed in this study could also originate from the nature of AML as a genetically heterogeneous disease and further assessment on the relationship between CYP1A1 MspI polymorphism and risk of AML subtypes might provide more instructive information. Additionally, gene-gene and gene-environment interactions should also be considered in the further investigations.

In summary, the results of the present meta-analysis suggest that variant C allele of CYP1A1 MspI polymorphism might have an association with increased AML risk among Asians. Further investigations are needed to confirm the conclusions.

## Competing interests

The authors declare that they have no competing interests.

## Authors' contributions

WZand ZC conceived of the study, and carried out the analysis of the literatures and drafted the manuscript. LZ and YW carried out the collection of the literatures. BZ helped with the statistical analysis and manuscript drafting. ZC and WZ conceived of the study, and participated in its design and coordination and helped to draft the manuscript. All authors read and approved the final manuscript.
